# Antioxidant Effect in Diabetic Peripheral Neuropathy in Rat Model: A Systematic Review

**DOI:** 10.3390/antiox13091041

**Published:** 2024-08-28

**Authors:** Noradliyanti Rusli, Chen Fei Ng, Suzana Makpol, Yin Ping Wong, Isma Liza Mohd Isa, Rabani Remli

**Affiliations:** 1 Department of Medicine, Faculty of Medicine, Universiti Kebangsaan Malaysia, Cheras 56000, Malaysia; p116652@siswa.ukm.edu.my; 2 Department of Neurology, Sunway Medical Centre, Subang Jaya 47500, Malaysia; ngchenf@sunmed.com.my; 3 Department of Biochemistry, Faculty of Medicine, Universiti Kebangsaan Malaysia, Cheras 56000, Malaysia; suzanamakpol@ppukm.ukm.edu.my; 4 Department of Pathology, Faculty of Medicine, Universiti Kebangsaan Malaysia, Cheras 56000, Malaysia; ypwong@ppukm.ukm.edu.my; 5 Department of Anatomy, Faculty of Medicine, Universiti Kebangsaan Malaysia, Cheras 56000, Malaysia; ismaliza.mohdisa@ukm.edu.my; 6 CÚRAM, SFI Research Centre for Medical Devices, School of Medicine, University of Galway, H91 TK33 Galway, Ireland

**Keywords:** diabetic peripheral neuropathy, antioxidants, systematic reviews

## Abstract

Oxidative stress is a contributing factor that leads to the vascular complications of diabetes mellitus. Diabetic peripheral neuropathy (DPN) is one of the microvascular complications with rising concern as the disease progresses despite strict glucose control and monitoring. Thus, there is an ongoing need for an early intervention that is effective in halting or slowing the progression of DPN where antioxidants have been proposed as potential therapeutic agents. This systematic review aims to evaluate the existing evidence on the antioxidant effect in DPN and provide insight on the role of antioxidants in the progression of DPN in a rat model. A comprehensive literature search was conducted on Web of Science, EBSCOhost, and Scopus to identify the effects and role of antioxidants in DPN. Data extraction was performed and SYRCLE’s risk of bias (RoB) tool was used for risk assessment. This systematic review was written following the PRISMA 2020 statements. From the literature search, 1268 articles were screened, and a total of 101 full-text articles were further screened before 33 were analyzed. These findings collectively suggest that antioxidants can play a crucial role in managing and potentially reversing the effects of diabetic neuropathy by targeting oxidative stress and improving nerve function.

## 1. Introduction

Diabetes mellitus is a chronic metabolic disorder characterised by elevated levels of glucose in the blood. Insufficient insulin production by the body or insulin resistance is the main factor contributing to the disease manifestation in patient with Type II diabetes mellitus (TIIDM) [1]. According to the International Diabetes Federation (IDF), approximately 463 million adults ages between 20 to 79 were living with diabetes worldwide, and this number was projected to rise to 629 million by 2045 if the current trends continue [2,3]. This global prevalence has reached epidemic proportions. Diabetes poses significant health challenges to both the healthcare system and society through the manifestation of macrovascular and microvascular complications of diabetes [4]. 

Diabetic peripheral neuropathy (DPN) is one of the microvascular complications of diabetes, affecting about 50% of diabetic patients. Most patients are already developing DPN at the time of their primary diagnosis [5]. Patients will normally develop somatic symptoms, including numbness, paraesthesia, hypo- or hyperalgesia, allodynia, and neuropathic pain [6,7,8]. The most typical symptom that can be observed in the patient is the development of stocking-glove pattern neuropathy where the patient has a vague disturbance of sensation in the feet as well as in the hand. The symptoms normally begin in the feet and progress proximally in a length-dependent manner due to the nature of the neurons [9,10]. The neuropathic pain can be very severe and debilitating and even disturb the normal routine and function of the patient. Some patients might also develop autonomic dysfunction, leading to disability and recurrent hospitalization [11]. Most of the time, patients do not identify the correlation of these symptoms to their neuropathic pain. These conditions not only significantly compromise the quality of life for those affected but also pose challenges in terms of timely diagnosis and effective management. 

In developing targeted interventions to reduce the diabetic neuropathy prevalence among diabetic patients, a more profound and thorough understanding of the complex association between diabetic mellitus and neuropathy is crucial. An increase in oxidative stress is one of the results of uncontrolled and persistent hyperglycemia and one of the main suspects contributing to the progression of neuropathy in diabetic patients [6]. Hyperglycemia in diabetic patients promotes auto-oxidation of glucose, causing excess production of advanced glycation end products (AGEs), which in turn activate the polyol pathway [12,13]. Aldose reductase, one of the main enzymes responsible for the polyol pathways, is abundantly expressed in the peripheral nerve, suggesting that these pathways are involved in the DPN pathogenesis.

A reduction in the number of enzymes involved in the antioxidant defense system such as glutathione (GSH), glutathione peroxidase (GPx), superoxide dismutase (SOD), and catalase (CAT) in diabetic patients might also have an impact on the progression of DPN [14,15]. This reduction causes an increase in free radical and reactive oxygen species (ROS), which concurrently increases the lipid peroxidation (LPO) process [16]. Neurons are known to have higher polyunsaturated fatty acids (PUFA) contents and are prone to excessive LPO during high free radical and oxidative stress conditions [17]. By-products of lipid peroxidation, including malondialdehyde (MDA) and 4-hydroxynonenal (4-HNE), are significantly increased in diabetic patients and are associated with neuronal loss in neurodegenerative disorders [18]. Elevated oxidative stress and heightened lipid peroxidation are implicated as destructive factors linked to the progression of β-cell dysfunction, insulin resistance, and impaired glucose tolerance, ultimately contributing to the pathogenesis of micro- and macrovascular complications of TIIDM, including DPN.

Recently, there has been emerging interest in the effects of antioxidants, specifically from natural products, on DPN using in vivo studies. Various animal models have been used to study the pathogenesis of DPN, specifically by using rats due to their susceptibility to diabetes-induced neuropathy. A few different methods are available for inducing DPN in rat animal models, either by using a chemical like Streptozotocin (STZ), a high-fat diet (HFD), or a combination of both [19]. These in vivo studies serve as a good screening platform for the candidates to treat DPN and further understand the pathogenesis of the disease and its progression. However, no information on these specific and reliable DPN animal models was acknowledged until recently. There is also a lack of information on the effects of various natural products and compounds with antioxidant properties on DPN despite the known role of oxidative stress in the pathogenesis of DPN. In the drug development process, it is important to have complete data on the potential candidates and their influence on the oxidative stress parameters as well as on the DPN-associated parameters. Thus, in this systematic review, a comprehensive understanding of the effects of the antioxidants on various parameters related to the pathogenesis of DPN is discussed. Details on the type of animal model used to represent DPN and the validation parameters on DPN used are also highlighted. Findings from previous studies also help to underscore the therapeutic potential of antioxidants in managing these complications, highlighting their ability to reduce oxidative stress, improve nerve function, and alleviate neuropathic pain.

## 2. Materials and Methods

This systematic review was conducted in accordance with the Preferred Reporting Items for Systematic Reviews and Meta-Analyses (PRISMA) guidelines 2020 and followed the PICOS framework (population, intervention, comparison, outcomes, and study design). Our literature review was registered in the international prospective register of systematic reviews, PROSPERO (https://www.crd.york.ac.uk/prospero/ (accessed on 21 March 2023)), with registration number CRD42023398467.

### 2.1. Search Strategy

We searched using the Web of Science, EBSCOhost, and Scopus electronic databases to identify all relevant articles related to the effects of various antioxidants (intervention) on diabetic peripheral neuropathy rats (population) in comparison to the vehicle-treated control rat. The behavioral, biochemical, and histopathology outcomes were noted, and only in vivo studies (study design) were included in this review. During the search, we used prespecified search engines for each database. Search strategies for different databases were discussed by NR, RR, and NCF with the following MeSH terms, antioxidant activity and diabetic neuropathy, as shown in Table 1. A systematic search of the literature was performed from 18 until 20 May 2022, and all data were pooled and kept in EndNote (Version 20) software (Clarivate, UK). An additional search was performed on 4 January 2024 using the same search strategy to include any recent articles in this systematic review. The search was also set to include only publications in the English language from the year 2000 onwards.

### 2.2. Selection Criteria and Eligibility Criteria

Three independent reviewers (NR, RR, and CFN) conducted a comprehensive review of titles, abstracts, and full-text articles and any discrepancies were resolved through discussion with other reviewers. Studies on diabetic neuropathy animal models with intervention with either plant extracts or compounds with antioxidant properties were included in this systematic review. Measurement of the antioxidant parameters, as well as diabetic peripheral neuropathy behavioral parameters, were also included in the inclusion criteria, with histopathological parameters as an added value. Interventions with drugs, in vitro studies, and no relevant outcome measures reported were excluded. Abstracts, review articles, meta-analyses, case reports, conference proceedings, and editorials/letters were also excluded from this study.

### 2.3. Data Extraction and Analysis

Data from the selected studies were extracted and input into Microsoft Excel 365 MSO (Version 2407) by NR, RR, and CFN to check for the inclusion and exclusion criteria based on the title and abstract. Studies in which the title and abstract fulfil the inclusion criteria were then advanced to full-text screening, where data on the DPN’s animal model, type of intervention, and predefined relevant outcomes were extracted.

### 2.4. Quality Assessment

**Figure 1:**
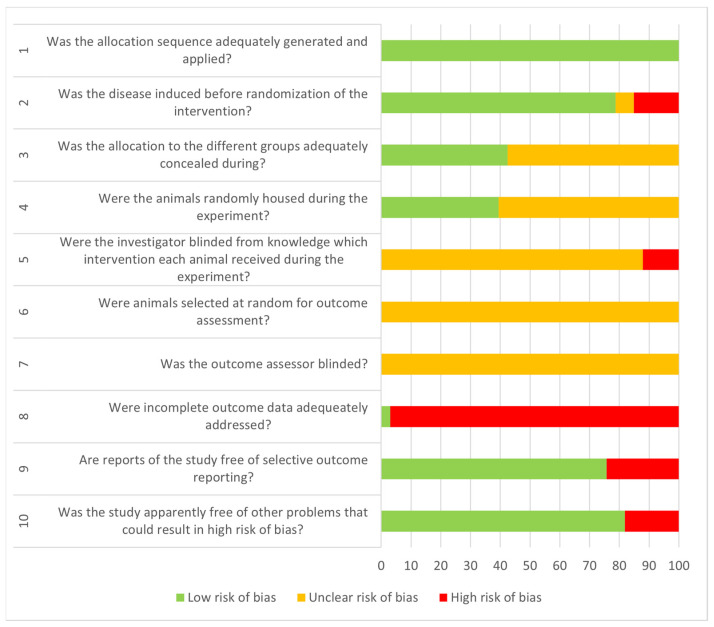
Result analysis for risk assessment using SYRCLE’s tool.

To ensure the quality of articles included in this systematic review, quality assessment was independently performed by NR and RR using SYRCLE’s risk of bias (RoB) tool for animal studies following the CAMARADES checklist for bias risk assessment [20]. This tool helps to systematically evaluate the risk of bias in various domains such as selection bias, performance bias, detection bias, attrition bias, reporting bias, and other biases using questions shown in Figure 1. The results of the assessment of the six domains were graded according to the presence of the item, where a ‘Y’ indicates compliance while an ‘N’ indicates non-compliance; ‘NA’ indicates that data are unavailable. To further analyze the assessment, a value of 1 was given to ‘Y’ and a value of 0 was given to ‘N’ and ‘NA’, and the data were again tabulated into a graph. Articles with high values indicate a low risk of bias (RoB), while low values indicate a high risk of bias (RoB). All decisions made during the resolution process, including initial assessments, points of disagreement, discussions held, and final consensus, were documented to provide a transparent record of the quality assessment process and support the reliability of the review’s findings.

## 3. Results

### 3.1. Study Selection

**Figure 2:**
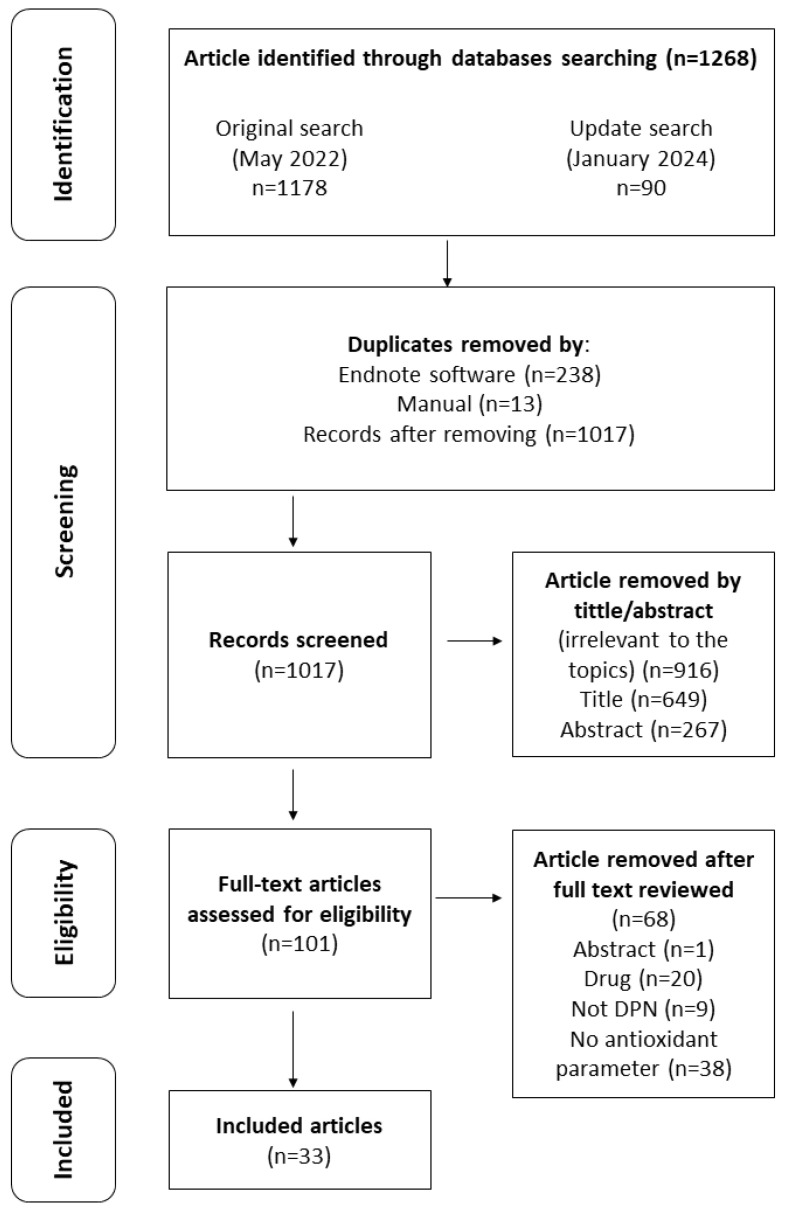
Flowchart of the process of literature search and extraction of studies meeting the inclusion criteria.

From the initial database search, a total of 1268 articles were obtained: 870 articles were from Scopus, 301 articles from Web of Science, and 97 articles from EBSCOhost. Duplicates were removed using EndNote (Version 20) software (Clarivate, UK) (*n* = 238), and an additional manual check (*n* = 12) was conducted before screening on the title and abstract were performed. During the title and abstract screenings, using an abstract screening form, 650 and 267 records were removed, respectively, as the records did not fulfil the inclusion criteria mentioned in Section 2.2. During the full-text articles assessment for eligibility, 101 records were screened and articles with no antioxidant parameter (*n* = 38) and used drugs (*n* = 20) for the intervention were excluded from this systematic review. Articles with no DPN animal model or those using models other than the DPN animal model (*n* = 9) and an abstract (*n* = 1) were also excluded from the study. At the end of the screening, a total of 33 records (Figure 2) were included in this systematic review, where the effects of various plant extracts and compounds with antioxidant properties on DPN were assessed and tabulated.

### 3.2. Description of the Studies

Out of 1268 articles, 33 records met the inclusion criteria to study the effects of antioxidants on the DPN animal models. All the studies used a DPN animal model, either Type I (*n* = 30) or Type II (*n* = 3) diabetes mellitus with a range of streptozotocin concentrations to induce the diabetic condition in the animal model. Two species of rats, weighing between 150 and 300 g, were used in the studies, with 15 records using Sprague Dawley (SD) rats and 18 records using Wistar rats, as shown in Table 2. Among all the interventions used, 9 of the interventions were plant or fungi extract and the remaining 24 of the interventions were active compounds; the concentrations used in these studies is shown in Table 2. The treatment duration for each of the studies are also summarized in Table 2, where the shortest treatment duration was 1 week [21] and the longest treatment duration was 48 weeks [22]. 

Various behavioral parameters were performed to confirm the development and progression of DPN in the animal model such as cold and hot plate study, formalin study, Hargreaves study, Randall–Selitto study, nerve conduction study, Von Frey filament study, tail flick study, as well as tail immersion study. These behavioral studies were performed as DPN involves more pain and increased thermal sensation. To measure the antioxidants properties, biochemical studies on the antioxidant parameters such as catalase enzyme (CAT), reduced glutathione (GSH), Glutathione S-transferases GST), glutathione peroxidase (GPx), lipid peroxidation (LPO), malondialdehyde (MDA), superoxide dismutase (SOD), total antioxidant content (TAC), nitric oxide (NO), Thioredoxin (Trx), and Thiobarbituric acid reactive substance (TBARS) were performed on either tissue or blood samples from the animal.

Other than the biochemical parameters on antioxidant level, ten records assessed the anti-inflammatory effects of the selected compound or extract, which include interleukin-1β (IL-1β), interleukin-6 (IL-6), insulin-like growth factor-1 (IGF-1), nuclear factor-κB (NF-κB), tumor necrosis factor-alpha (TNF-α), and transforming growth factor-β (TGF-β). Only 13 records conducted an analysis on the histopathological parameters of DPN, where 9 records performed Haematoxylin and Eosin (H&E) staining on the sciatic nerve, 1 record each performed H&E staining on brain and skin, respectively, and the other 2 records performed intraepidermal nerve fiber density (IENFD) staining on the skin. Additionally, one record performed electron microscopy on the sciatic nerve to observe the effect of the compound or extract on the morphology of the sciatic nerve in detail.

### 3.3. Animal Model for DPN

As DPN progression occurs alongside the development of diabetes in patients and animals, the development of diabetes in the animal model was first observed in all the studies. All the studies used streptozotocin (STZ) with a range of concentrations, starting with 30 mg/kg (*n* = 1) [23] and increasing through 35 mg/kg (*n* = 2) [21,22], 40 mg/kg (*n* = 1) [24], 45 mg/kg (*n* = 3) [25,26,27], 50 mg/kg (*n* = 3) [11,28,29], 55 mg/kg (*n* = 9) [1,3,5,30,31,32,33,34,35], 60 mg/kg (*n* = 8) [6,36,37,38,39,40,41,42], 65 mg/kg (*n* = 5) [43,44,45,46,47], and 80 mg/kg (*n* = 1) [48].

In all the studies, STZ was injected into the rats via intraperitoneal (ip) injection except for two studies [25,29], during which STZ was injected via intravenous (iv) injection. In addition, food was removed from the cage as the animal must be fasting for 12 h prior to the STZ injection. In five of the studies, 5% glucose solution was supplemented to the rats overnight post-STZ injection to prevent hypoglycemia in the animal due to the effects of STZ [5,11,25,29,44]. One of the studies also used nicotinamide (NAD) (230 mg/kg/ip) together with STZ to induce diabetic conditions in the animal [45]. As mentioned above, most of the studies used the Type I diabetic animal model, while three of the studies used the Type II diabetic animal model [21,22,23]. In the Type II diabetic animal model, the animal was supplemented with a high-fat diet to induce obesity and to mimic Type II diabetic conditions.

**Table 2:** Effect of antioxidants on diabetic peripheral neuropathy (DPN). Animal model, plant extract/compound and outcomes of studies included in the systematic review.

Authors, Year	Animal Model	Plant Extract. Compound	Treatment Duration	Rat Species. Age/Weight	Behavioral Parameter	Biochemical Parameter	Histopathological Parameter
Adki 2021 [30]	Type 1 DM STZ (55 mg/kg/ip)	Paeonol 50, 100 and 200 mg/kg/day	4 w	male Sprague-Dawley rats 180–220 g	↑ HP, MNCV, SNCV, RS, TI, VF	Sciatic nerve: ↑ GSH, SOD, CAT ↓ MDA	Sciatic nerve H&E
Al-Rejaie 2015 [36]	Type 1 DM STZ (60 mg/kg/ip)	Naringenin 25 and 50 mg/kg/po/day after 2 w become diabetic	5 w	Wistar albino rat 250–290 g	↑ RS, TF	Sciatic nerve: ↑ GSH, SOD, CAT, GPx, NGF, IGF ↓ TBARS, IL-1β, IL-6, TNF-α	Sciatic Nerve H&E
AlSharari 2014 [43]	Type 1 DM STZ (65 mg/kg/ip)	Morin 15 or 30 mg/kg/po/day after 3 w become diabetic	5 w	male Wistar albino rats 260–285 g	↑ RS, TF	Sciatic nerve: ↑ NGF, IGF-1, GSH, SOD, CAT ↓ TNF-α, IL-1β, IL-6, TBARS	none
Archana 2022 [29]	Type 1 DM STZ (50 mg/kg/iv)	250, 500 mg/kg/d/oral *Tinospora cordifolia* after 1 w become diabetic	7 w	male Albino Wistar rats 180–220 g	↑ HP, TI (warm and cold), Rotarod	Sciatic nerve: ↑ GSH, CAT, SOD ↓ MDA	none
Balaha 2018 [5]	Type 1 DM STZ (55 mg/kg/ip) 5% glucose solution to drink overnight of STZ injection	Phloretin 50 or 25 mg/kg/po/day after 4 w become diabetic	4 w	male Wistar rats 150–200 g	↑ HP, TI (warm and cold)	Sciatic nerve: ↑ GSH, SOD ↓ TNF-α, IL-6, MDA	Sciatic Nerve H&E, toluidine blue
Baluchnejadmojarad 2010 [37]	Type 1 DM STZ (60 mg/kg/ip)	Silymarin 100 mg/kg/ip/day	8 w	male Wistar albino rats 240–290 g	↓ FT ↑ HP, MNCV	↑ SOD ↓ MDA	none
Bana 2023 [6]	Type 1 DM STZ (60 mg/kg/ip)	*R. cordifolia* 200 and 400 mg/kg/po after 4 w become diabetic	4 w	male Wistar rats 150–300 g	↑ HP, TF	Sciatic nerve: ↑ GSH, SOD, CAT ↓ TBARS	Brain and Sciatic nerve H&E
Bin-Jaliah 2013 [44]	Type 1 DM STZ (65 mg/kg/ip) 5% glucose solution to drink overnight of STZ injection	Vitamin E 300 mg/kg/im/3x per week	4 w	male Wistar rats 200–250 g	↓ NCV	Sciatic nerve: ↓ MDA, ↑ GPx	none
Comelli 2009 [38]	Type 1 DM STZ (60 mg/kg/ip)	*Cannabis sativa* extract 15 or 30 mg/kg/po/day after 28 days from STZ injection	8 days	male Wistar rats 200–220 g	↓ H ↑ VF	Liver: ↓ MDA ↑ GSH Sciatic nerve tissue: ↑ NGF	none
Cui 2008 [1]	Type 1 DM STZ (55 mg/kg/ip)	Grape seed proanthocyanidin extract (GSPE) 250 mg/kg/day	24 w	male Wistar rats 200–220 g	↑ NCV, VF	Sciatic nerve: ↓ MDA ↑ SOD	Sciatic Nerve H&E
Dhaliwal 2020 [3]	Type 1 DM STZ (55 mg/kg/ip)	Ferulic acid (FA) 25, 50, and 100 mg/kg/po/day after 4 w become diabetic	4 w	male Sprague-Dawley rats 250–300 g	↑ CP, HP, MNCV, RS, VF	Sciatic nerve: ↑ NGF, SOD, GSH, CAT ↓ TNF-α, IL-1β, LPO	none
El-Baz 2020 [11]	Type 1 DM STZ (50 mg/kg/ip) 5% glucose solution overnight of STZ injection	*Dunaliella salina* powder 100 or 200 mg/kg after 2days become diabetic	4 w	male Wister albino rats 150–200 g	↓ HP	Blood: ↑ SOD, TAC, CAT Brain tissue: ↑ Trx, ↓ TNF-α, and IL-6.	Brain H&E
Fink 2020 [23]	Type 2 DM high-fat diet after 8 w, STZ (30 mg/kg/ip)	Mito-q 0.93 g/kg/po/day	12 w	male Sprague-Dawley rats 10–11w	↑ MNCV, SNCV	↓ LPO	IENFD
Kamdi 2021 [21]	Type 2 DM high-fat diet after 2 w, STZ (35 mg/kg/ip)	Apple peel extract (APE) 100, 200 and 400 mg/kg/po/day after 2 w become diabetic	7 days	male Sprague-Dawley rats 160–180 g	↑ H	↑ GSH ↓ CAT, MDA	skin H&E
Kandhare 2012 [31]	Type 1 DM STZ (55 mg/kg/ip)	Naringin 20, 40 and 80 mg/kg/po/day after 4 w become diabetic	4 w	adult male Wistar rats 150–200 g	↑ MNCV, RS, TI, VF	↑ SOD ↓MDA, NO, TNF-α	none
Kiasalari 2017 [39]	Type 1 DM STZ (60 mg/kg/ip)	Diosgenin 40 mg/kg/po/day after 8 days become diabetic	5 w	male albino Wistar rats 210–250 g	↓ FT ↑ RS, TI	Sciatic nerve: ↓ MDA ↑ SOD, CAT blood: ↓ TNFα, NF-κB, IL-1β	none
Kim 2013 [48]	Type 1 DM STZ (80 mg/kg/ip) 2x in two conservative days	Berberine 5, 10, and 20 mg/kg/ip/bid after 4 w become diabetic	14 days	male Sprague-Dawley rats 170–190 g	↑ CP, VF	Liver: ↓ MDA, SOD, GSH, CAT	none
Kishore 2017 [45]	Type 1 DM STZ (65 mg/kg/ip) Nicotinamide (NAD) (230 mg/kg/ip)	Bacopa monnieri alcohol extract (BA) 100, 200 and 400 mg/kg/po/day Bacosine (BS) 5 and 10 mg/kg after 60 days	30 days	male Wistar rats 260–280 g	↑ HP, MNCV, RS, TI, VF	Sciatic nerve: ↑ SOD, GSH ↓ TNF-α, TGF-β, IL-1β	none
Koneri 2014 [25]	Type 1 DM STZ (45 mg/kg/iv) 5% glucose solution overnight of STZ injection	Saponins of *Momordica cymbalaria* (SMC) (100 mg/kg/po/day on day 3	30 days	male Wistar rats 200–250 g	↓ HP, TF	Sciatic nerve: ↓ SOD, CAT, MDA	none
Kuhad 2009 [26]	Type 1 DM STZ (45 mg/kg/ip)	Tocotrienol 25, 50 and 100 mg/kg/oral/day after 4 w	4 w	male Wistar rats 220–260 g	↑ HP, RS, TI, VF	Sciatic nerve: ↑ SOD, CAT, ↓ TNF-α, TGF-β, IL-1β, MDA, NO	none
Kumar 2007 [32]	Type 1 DM STZ (55 mg/kg/ip)	Resveratrol 10, 20 mg/kg/ip/day after 6 w become diabetic	2 w	male Sprague-Dawley rats 250–270 g	↑ MNCV, TI, VF	↑ CAT ↓ MDA, peroxynitrite, DNA fragmentation (TUNEL)	none
Lee 2018 [40]	Type 1 DM STZ (60 mg/kg/ip)	Alpha lipoic acid 100 mg/kg	12 w and 24 w	male Sprague-Dawley rats 180–200 g	not significant RS, TF, VF	Blood: ↑ CAT, SOD GSH	Sciatic nerve toluidine blue, IENFD
Liu 2014 [46]	Type 1 DM STZ (65 mg/kg/ip)	Zinc supplementation after 4w become diabetic	4 w	male Sprague-Dawley rats	↑ MNCV, RS	Sciatic nerve: ↓ MDA	Immunohisto staining Metallothionein
Liu 2010 [28]	Type 1 DM STZ (50 mg/kg/ip)	Tanshinone IIA 20, 50, 100 mg/kg after 4 days become diabetic	4 w	male Sprague–Dawley albino rats 180–220 g	↑ HP, MNCV, VF	Sciatic nerve: ↓ MDA ↑ SOD, CAT	none
Najafi 2017 [47]	Type 1 DM STZ (65 mg/kg/ip)	cerium oxide nanoparticles 65/85 mg/kg/po after 8 w become diabetic	8 w	male Wistar rats 180–250 g	↑ HP	↓ MDA, ↑ TAC	none
Ranjithkumar 2013 [24]	Type 1 DM STZ (40 mg/kg/ip)	aqueous *Tribulus terristris* 100 and 300 mg/kg/po after 4 weeks diabetic	4 w	male Wistar rats 225–250 g	↑ CP, FT, HP	↑ SOD, CAT, GSH, ↓ MDA, TNF-α, IL-1β	none
Rashedinia 2020 [41]	Type 1 DM STZ (60 mg/kg/ip)	Syringic acid 25, 50, and 100 mg/kg	6 w	male Sprague-Dawley rats 220–240 g	↑ Passive Avoidance Test, rotarod test	Brain: ↓ GSH, MDA not significant CAT, SOD	Sciatic H&E
Sharma 2006 [33]	Type 1 DM STZ (55 mg/kg/ip)	Trolox 10 and 30 mg/kg/ip after 6w become diabetic	2 w	male Sprague-Dawley rats 250–270 g	↑ MNCV, TI	Sciatic nerve: ↓ MDA ↑ SOD, CAT	none
Suryavanshi 2020 [34]	Type 1 DM STZ (55 mg/kg/ip)	Escin 5, 10 and 20 mg/kg/po/day after 6w become diabetic	4 w	male Sprague-Dawley rats 180–230 g	↑ HP, MNCV, RS, TI, VF	Sciatic nerve: ↓ MDA ↑GSH, SOD	Sciatic H&E
Tian 2016 [35]	Type 1 DM STZ (55 mg/kg/ip)	Rutin 5, 25 and 50 mg/kg/ip/day after 3 w become diabetic	2 w	male Sprague-Dawley rats 200–240 g	↑ CP, HP, MNCV, SNCV, VF	Sciatic nerve: ↑ SOD, GST, GPx ↓ MDA Blood: ↓ IL-6, TNF-α, NF-κB	none
Tiwari 2011 [27]	Type 1 DM STZ (45 mg/kg/ip)	Emblica officinalis aqueous extract 250, 500 and 1000 mg/kg/po/day after 4w become diabetic	4 w	male Wistar rats 220–260 g	↑ HP, RS, TI, VF	Sciatic nerve: ↓ LPO ↑ GSH, SOD ↓ IL-1β, TNF-α Blood: ↓ IL-1β, TNF-α	none
Yang 2015 [42]	Type 1 DM STZ (60 mg/kg/ip)	Tang Bi Kang (TBK) 4.28, 8.56, 17.12 g/kg/po/day	4 w	male Wistar rats 180–220 g	↑ NCV, TF	↓ MDA ↑ SOD, GSH	Sciatic H&E
Zhou 2012 [22]	Type 2 DM high-fat diet, STZ (35 mg/kg/ip)	Trigonelline 40 mg/kg/po/day	48 w	male Wistar rats 180–220 g	↑ NCV, TI	↓ MDA ↑ SOD	Electron microscopy of sciatic nerve

Abbreviation used: bid: bis in die/twice a day; CAT: catalase enzyme; CP: cold plate; DM: diabetic mellitus; DNA: Deoxyribonucleic acid; FT: formalin test; kg: kilogram; ip: intraperitoneal; po: per oral; w: week; g: gram; GSH: reduced glutathione; GST: Glutathione S-transferases; GPx: glutathione peroxidase; H: Hargreaves test; HP: hot plate; H&E: Hematoxylin and Eosin staining; IENFD: Intraepidermal nerve fiber density staining; iv: intravenous; IL-1β: Interleukin-1β; IL-6: Interleukin-6; IGF-1: insulin-like growth factor-1; LPO: lipid peroxidation; MDA: malondialdehyde; mg: milligram; MNCV: motor nerve conduction velocity; NCV: nerve conduction velocity; NF-κB: nuclear factor-κB; NGF: nerve growth factor; SNCV: sensory nerve conduction velocity; SOD: superoxide dismutase; STZ: streptozotocin; RS: Randall–Selitto test; TAC: total antioxidant content; TI: tail immersion; TF: tail flick; TNF-α: tumor necrosis factor-alpha; TGF-β: Transforming growth factor-β; NO: nitric oxide; TUNEL: terminal deoxynucleotidyl transferase dUTP nick end labelling; Trx: Thioredoxin; TBARS: Thiobarbituric acid reactive substance; VF: Von Frey filament test; ↓: decrease or down-regulated; ↑: increase or up-regulated.

### 3.4. Effects of Compounds or Extracts on DPN Behavioural Parameters

**Figure 3:**
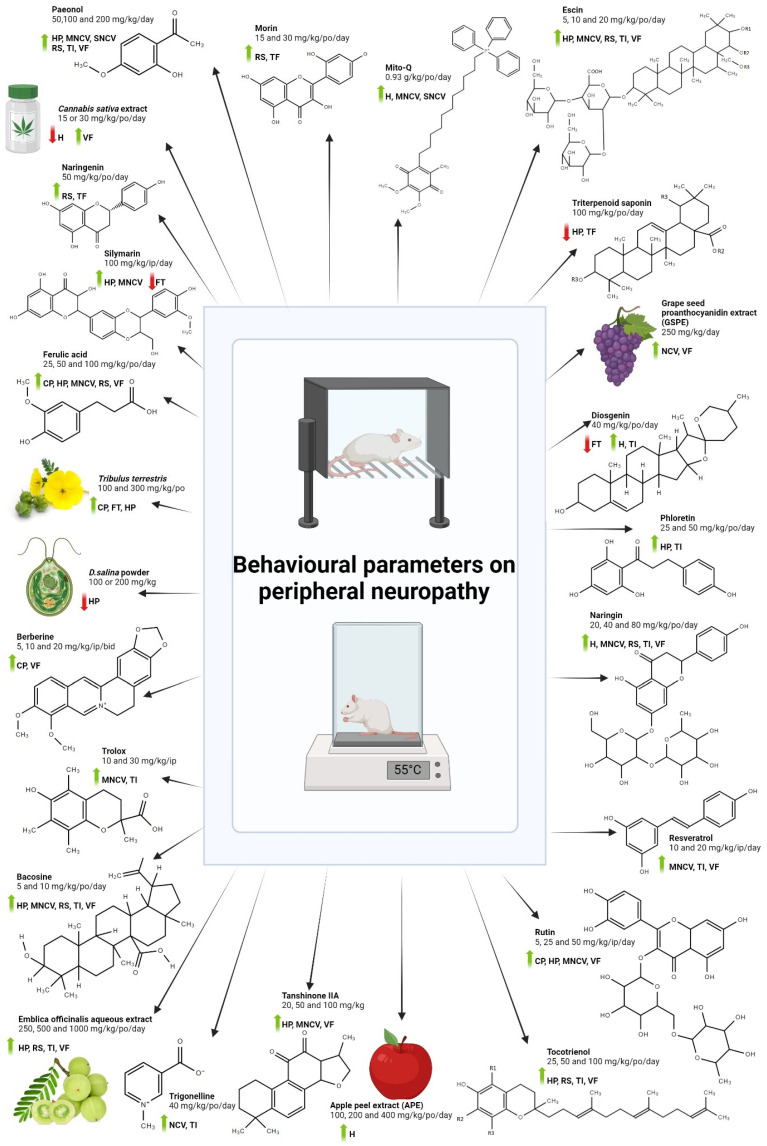
Effect of various plant extract/compounds on behavioral parameters on diabetic peripheral neuropathy (DPN). Cold plate (CP), Hargreaves test (H), Hot plate (HP), formalin test (FT), motor nerve conduction velocity (MNCV), nerve conduction velocity (NCV), sensory nerve conduction velocity (SNCV), Randall–Selitto (RS), Tail immersion (TI), Tail flick (TF), and Von Frey filament study (VF) are the list of behavioral parameters being measured in the selected studies. Green arrow indicates improvement in the DPN symptoms while red arrow indicates worsen of DPN symptoms. Create with BioRender.com, accessed on 2 August 2024.

To observe the development of DPN in the animal model, behavioral studies were performed and the effect of various compounds and extracts on the DPN progression was monitored. As shown in Figure 3, improvement in nerve conduction studies, thermal allodynia, as well as hyperalgesia were seen with most of the treatments. However, alpha lipoic acid did not show a significant reduction in these behavioral parameters. For a study on syringic acid, a passive avoidance test and rotarod test were used for the DPN behavioral parameters, and an increase in the response during both tests was observed.

### 3.5. Effects of Compounds or Extracts on DPN Biochemical Parameters

**Figure 4:**
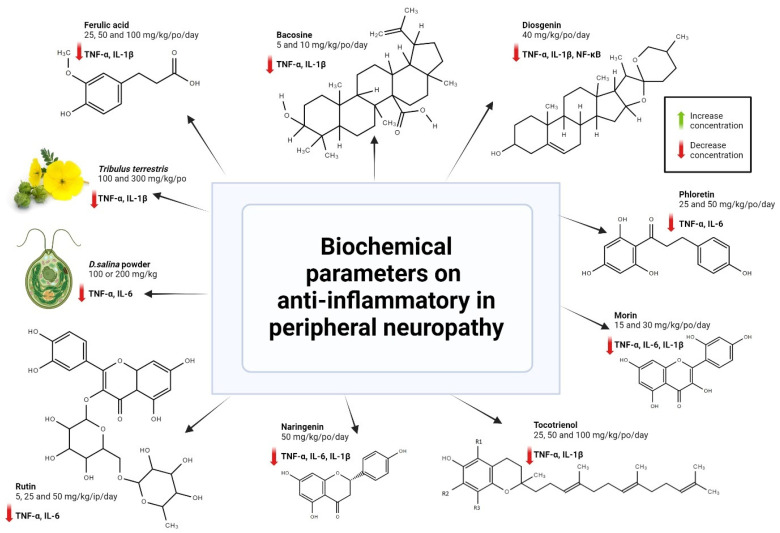
Effect of various plant extract/compounds on inflammatory parameters on diabetic peripheral neuropathy (DPN). Created with BioRender.com, accessed on 2 August 2024.

To confirm the antioxidant effects of the compounds and plant extract, biochemical parameters on the tissues or blood samples of the rat treated group were measured. A significant increase in the antioxidant enzyme level (SOD, CAT, GSH) as well as a significant reduction in the LPO and its by-products (MDA) were observed in the treatment group compared to the control, as shown in Table 2. Some of the studies also include anti-inflammatory parameters, where a significant reduction in the anti-inflammatory mediator’s level was shown in the treated group compared to the control group, as shown in Figure 4.

### 3.6. Histological Parameters for DPN

Other than behavioral parameters, histopathological parameters can also be used to monitor the development and progression of DPN in animal models. The most common histopathological parameter measure among the studies was H&E staining on the sciatic nerve, where a significant increase in the neuronal count and a significant decrease in neuropathic lesions were monitored following treatment with paeonol, naringenin, phloretin, grape seed proanthocyanidin extract, alpha lipoic acid, syringic acid, escin, and Tang Bi Kang [1,5,30,34,36,40,41,42]. H&E staining on the brain and skin sections also showed a decrease in neuropathic lesions, an increase in the neuronal count, and a decrease in inflammatory infiltration following *Dunaliella salina* powder and Apple peel extract [11,21]. Most recently, IENFD on the skin sample was used for the detection of DPN and a significant increase in the IENFD count was identified with the treatment of Mito-q and alpha lipoic acid [23,40].

### 3.7. Assessment of the Risk of Bias (RoB) Tool for Animal Studies

From the assessment, the highest value for the risk of bias (RoB) tool was 7 (*n* = 1) while the lowest value was 1 (*n* = 1) [1,23]. Other records had a value of 6 (*n* = 5), 5 (*n* = 8), 4 (*n* = 11), 3 (*n* = 3), and 2 (*n* = 4), as shown in Table 3. From Figure 1, all the records fulfil the requirement for question 1 on the allocation sequence. About 78.8%, 75.8%, and 81.8% of the records had a low risk of bias on questions 2, 9, and 10, respectively. Information on the blinding of the study or on randomization for the outcome assessment is not available.

## 4. Discussion

There is an urgent need to find a specific treatment despite symptomatic relief of DPN, and oxidative stress has been considered as a great potential therapeutic target. Our systematic review highlighted possible natural candidates with antioxidant properties and their effects on DPN behavioral parameters in rat animal models. In most of the studies, an increase in the antioxidant enzymes and reduction in the oxidative stress byproduct were identified post-treatment, which led to the improvement in the neuropathy behavioral parameters in the rat animal models, as shown in Table 2. Some of the studies also correlate antioxidant properties with the anti-inflammatory effects of the compound on the DPN rat animal model.

### 4.1. Variability in Study Design

To date, no established animal model on DPN has been documented, and various models have been used to study pathogenesis as well as to screen for potential candidates for DPN treatment. Based on the records selected, the development of the diabetic condition in the rat animal model, either Type I or Type II DM, were first established in the animal model and further validated using behavioral tests to confirm the development of DPN in the animal model. Most of the studies (*n* = 30) utilized a Type I DM animal model to represent DPN. In these studies, rats were administered varying concentrations of STZ ranging from 30–80 mg/kg/injection. STZ is a widely used antibiotic that selectively destroys pancreatic beta cells, leading to a reduction in insulin production and the development of diabetes in rodents [49]. The Type I DM state can be induced either by a high single dose of STZ (<65 mg/kg), leading to rapid and extensive β-cell destruction, or by multiple low doses of STZ, causing partial β-cell damage, triggering an inflammatory response and ultimately resulting in further loss of β-cell activity [19]. Both approaches create a condition that closely mirrors the pathogenesis of Type I DM in humans.

Recently, research has shown that Type II DM animal models can be more effective in studying neuropathy [50]. It was found that both diet-induced obesity and hyperglycemia in rodents led to the development of neuropathy, suggesting that hyperglycemia alone does not drive this condition. In addition, the STZ induced animal model has been criticized as it lacks the morphological changes in the peripheral nerve [51]. It is also highlighted that the use of Type II DM rodents in modelling neuropathy focusing on diabetes-induced neuropathic pain has become a practice recently. These studies collectively support the use of Type II DM animal models in understanding and potentially treating neuropathy. From the records, there are two species of rats that have been used for mimicking DPN conditions in animal models: Spraque-Dawley rats (*n* = 13) and Wistar rats (*n* = 20). According to a study on different rat strains developing diabetic retinopathy and neuropathy, both strains showed significant tactile allodynia in peripheral nerves [52]. However, 5 out of 20 studies using Wistar rats had worse symptoms in the behavioral studies after treatment with antioxidants. Two of these studies used the formalin test, which is normally used to observe analgesic properties of compounds. Sprague-Dawley and Wistar rats also show similar IENFD in the hindpaw footpad, which correlates with sensory nerve conduction velocity changes in neuropathy models [53]. In addition, most of the records used male rats for the study due to their hormonal stability compared to female rats, whose hormonal fluctuations can introduce additional variability in experimental results [54]. All the studies implemented different rat models to induce DPN. There is no established animal model specifically designed to study DPN. Future studies should design and established a DPN animal model to produce a robust outcome.

To confirm the development of DPN in the animal model, a minimum of two behavioral studies or more were performed in most of the records, as shown in Table 2. The behavioral studies were used to assess the symptoms of DPN in the animal model, including thermal hyperalgesia and mechanical allodynia [55]. Nerve conduction studies, including motor and sensory nerve conduction studies, have also been used to investigate diabetic peripheral neuropathy in rodent animal models. Studies have revealed early reductions in nerve blood flow and conduction deficits in diabetic rats [56,57]. Nerve conduction studies, including motor and sensory nerve conduction studies, are valuable tools for assessing peripheral neuropathy in rodent models. It was demonstrated that the reliability of a minimally invasive motor nerve conduction study in rats provided a potential method for assessing motor nerve function in these models [58]. This is particularly relevant in studying diabetic peripheral neuropathy, chemotherapy-induced peripheral neuropathy, and human immunodeficiency virus-associated sensory neuropathies [59]. In addition, the use of nerve conduction assessments in nonclinical species further underscores the importance of these studies in evaluating the development of peripheral neuropathy in animal models [60].

Most recently, the European Federation of Neurological Societies has updated better recommendations for DPN diagnosis by performing quantification of the IENFD on a skin biopsy from the distal leg of the suspected patient [61]. This quantification can also be implied in the detection of DPN in animal models, where two of the studies used EINFD to confirm the development of DPN. Research has consistently shown that the density of intraepidermal nerve fibers is a reliable marker for diagnosing DPN. The research found a significant reduction in IENFD in patients with DPN, with a cutoff value of 10.1 fibers/mm for diagnosis [62,63]. Another study provided further support to the use of IENFD, comparing it to corneal confocal microscopy and finding comparable diagnostic efficiency [64]. Thus, performing this IENF density quantification in the study, together with the behavioral studies, is one of the recent important parameters to be explicitly included in understanding the effectiveness of potential treatment of DPN. However, from the selected articles, a lack of morphological studies on the affected nerve was observed despite the outcomes of the behavioral parameters, where only 10 out of 33 articles conducted histopathological studies, and only 2 conducted IENFD quantification.

### 4.2. Methodological Quality

In assessing the risk of bias (RoB) tool for animal studies in this systematic review, SYRCLE’s risk of bias (RoB) tool for animal studies, following the CAMARADES checklist for bias risk assessment, was used. Based on the results in Table 3, no reports on the practice of blinding in the studies selected can be observed. This is also supported by a study highlighting the low awareness and use rates of ARRIVE guidelines among medical researchers, suggesting the need for specific measures to promote and popularize the use of the guidelines [65]. The SYRCLE risk of bias tool as well as the ARRIVE guideline for animal studies are important tools for assessing and improving the methodological quality of animal studies [66]. The ARRIVE guidelines help to ensure clear and detailed documentation, making research articles easier to understand and aiming to make studies more transparent and reproducible [67]. The guidelines also emphasized the need for widespread adoption and empirical testing of these tools to enhance the efficiency of translating animal research into clinical practice [20,68].

### 4.3. Effect of Antioxidant on DPN Animal Model

**Figure 5:**
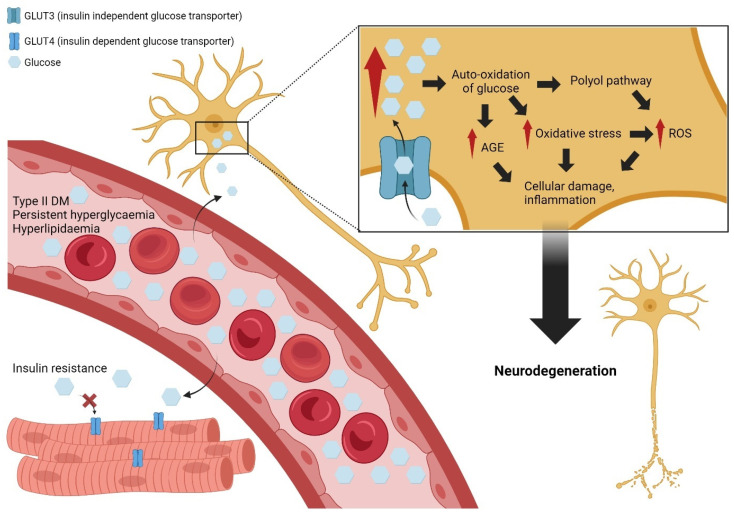
Effect of various plant extract/compound on inflammatory parameters on diabetic peripheral neuropathy (DPN). Red arrow indicates increase in the level of respective parameters. Create with BioRender.com, accessed on 2 August 2024.

Diabetic peripheral neuropathy is a complex condition with multifactorial pathogenesis. Key factors include changes in polyol pathway flux, oxidative stress, non-enzymatic protein glycation, and endothelial dysfunction, all of which contribute to reduced nerve blood flow and deficits in neurotrophic factors [69]. Diabetes-associated persistent hyperglycaemia is identified when there is a prolonged increase in blood glucose. In Type II DM, patients also experience hyperlipidemia and insulin resistance, which then lead to further increase in the blood glucose level [70,71]. Because of insulin resistance, glucose cannot be transported into the cell via insulin-dependent glucose transporters. However, an insulin-independent glucose transporter (GLUT3) is constitutively expressed in nerve cells regardless of insulin levels or insulin resistance [72]. This condition allows a high amount of glucose to be transported into the nerve cells and leading to the activation of various cellular signaling cascades, including polyol pathways, advanced glycation end (AGE) products formation pathways, as well as hexosamine pathways [73,74]. Activation of these pathways can generate reactive oxygen species (ROS), activate protein kinase C (PKC), and increase the production of AGEs, all of which contribute to cellular damage and dysfunction, specifically in nerve cells, as summarized in Figure 5.

From the selected studies, most of the natural compounds and extracts led to a significant reduction in the DPN symptoms monitored via the behavioral parameters in the animal models of DPN, as shown in Figure 3. Antioxidants have shown promise in improving neuropathy conditions, either in chemotherapy-induced peripheral neuropathy (CIPN) or DPN, where antioxidants such as N-acetylcysteine, α-lipoic acid, and vitamin E can alleviate neuropathy by inhibiting oxidative stress and neuroinflammation [75]. Antioxidants like paeonol, Mito-Q, Escin, naringin, rutin, bacosine, and ferulic acid improve the nerve conduction study via in vivo studies, and a similar pattern can be seen in the other behavioral parameters like hot or cold plate, Von Frey filament study, tail flick, and the Randall–Selitto test [3,23,31,35,45]. Improvement in DPN symptoms agrees with the improvement in the antioxidant enzyme level post-intervention, which indicates that there are correlations between the antioxidant enzyme level and improvement in the behavioral parameters for DPN. In most of the studies, an increase in enzymes like SOD, CAT, and GSH, which are responsible for the scavenging of oxidative stress as well as free radical in the cells, was observed after treatment with various natural antioxidants, as shown in Table 2. Other than that, reduction in the enzyme for lipid peroxidation and its by-product (MDA) can also be seen after the intervention with potential antioxidants.

Based on this systematic review, it can be said that most of the studies explore the potential of individual natural compounds and extracts without the use of any combined therapy or polypharmacy normally seen in diabetic patients with multiple comorbidities [76]. Future studies should investigate their ability to halt and reverse the progression of DPN without any interactions with current diabetic medications, and this might support their potential as candidates for DPN treatment in the future. In addition, potential biomarkers that are specific to DPN should also be used to further confirm and elaborate on the effects of these natural compounds and extracts in treating DPN. Neurofilament light chain and nerve growth factor are emerging as important biomarkers in the study of DPN and should be included in future DPN related studies [77]. Furthermore, these promising candidates for DPN treatment should be translated into clinical studies and detail dosing studies. Finally, studies concerning the timing of intervention relative to disease progression should also be completed in the future.

## 5. Conclusions

DPN is responsible for 50 to 75% of non-traumatic amputations in diabetic patients, and its prevalence is estimated to increase, thereby contributing to low quality of life as well as being a huge burden on healthcare systems. By understanding the pathogenesis of the disease, a specific and targeted intervention can be introduced to halt and slow the progression of DPN in patients. From this systematic review, we showed all the possible natural compounds as well as natural extracts that had the potential to help in improving DPN by reducing hyperglycemia-induced oxidative stress as well as by increasing the enzymes involved in the oxidative stress protective mechanisms. Since the use of ARRIVE guidelines in reporting in vivo studies is not widespread among researchers, some of the information required is not available from the articles. More awareness among researchers is needed to improve reporting in research articles, thus helping in improving data collection for future systematic reviews and meta-analyses. In conclusion, antioxidant therapy represents a promising avenue for the treatment of DPN, with the potential to significantly improve outcomes by targeting the oxidative stress pathway. Further research in this field is essential to translate these findings from animal models into clinical practice, benefiting individuals suffering from diabetic neuropathy. Future studies should focus on optimizing antioxidant treatment regimen dosages and elucidating the molecular mechanisms involved in their neuroprotective effects.

## Figures and Tables

**Table 1:** Search strategies for different databases were used for the literature search.

Database	Search Strategy
Web of Science	(((AB = (antioxidant activity)) AND AB = (diabetic neuropathy)) NOT AB = (in vitro)) NOT AB = (systematic review meta-analysis)
EBSCOhost	AB antioxidant activity AND AB diabetic neuropathy NOT AB in vitro NOT AB (systematic review or meta-analysis)
Scopus	(TITLE-ABS-KEY (antioxidant AND activity) AND TITLE-ABS-KEY (diabetic AND neuropathy) AND NOT TITLE-ABS-KEY (in AND vitro) AND NOT TITLE-ABS-KEY (systematic AND review AND meta AND analysis))

Abbreviation used depends on the database: AB: Abstract; ABS: Abstract; KEY: Keywords.

**Table 3:** Risk assessment using SYRCLE’s tool for bias risk assessment where a ‘Y’ with green color code indicates compliance, ‘N’ with red color code indicates non-compliance, and ‘NA’ with yellow color code indicates that data are unavailable.

Authors, Year	1	2	3	4	5	6	7	8	9	10	Total
Adki 2021 [30]	Y	Y	NA	NA	NA	NA	NA	N	Y	Y	4
Al-Rejaie 2015 [36]	Y	Y	NA	NA	NA	NA	NA	N	Y	Y	4
AlSharari 2014 [43]	Y	Y	NA	NA	NA	NA	NA	N	Y	Y	4
Archana 2022 [29]	Y	Y	Y	Y	NA	NA	NA	N	Y	Y	6
Balaha 2018 [5]	Y	Y	NA	Y	N	NA	NA	N	Y	Y	5
Baluchnejadmojarad 2010 [37]	Y	Y	NA	NA	N	NA	NA	N	Y	Y	4
Bana 2023 [6]	Y	Y	Y	Y	NA	NA	NA	N	Y	Y	6
Bin-Jaliah 2013 [44]	Y	NA	NA	NA	N	NA	NA	N	Y	Y	3
Comelli 2009 [38]	Y	Y	Y	NA	NA	NA	NA	N	Y	Y	5
Cui 2008 [1]	Y	Y	Y	Y	N	NA	NA	Y	Y	Y	7
Dhaliwal 2020 [3]	Y	Y	NA	NA	NA	NA	NA	N	N	N	2
El-Baz 2020 [11]	Y	Y	NA	NA	NA	NA	NA	N	N	N	2
Fink 2020 [23]	Y	NA	NA	NA	NA	NA	NA	N	N	N	1
Kamdi 2021 [21]	Y	Y	NA	NA	NA	NA	NA	N	Y	Y	4
Kandhare 2012 [31]	Y	Y	Y	NA	NA	NA	NA	N	Y	Y	5
Kiasalari 2017 [39]	Y	N	NA	NA	NA	NA	NA	N	Y	Y	3
Kim 2013 [48]	Y	Y	Y	Y	NA	NA	NA	N	N	N	4
Kishore 2017 [45]	Y	Y	NA	NA	NA	NA	NA	N	N	N	2
Koneri 2014 [25]	Y	Y	NA	NA	NA	NA	NA	N	Y	Y	4
Kuhad 2009 [26]	Y	Y	NA	NA	NA	NA	NA	N	N	N	2
Kumar 2007 [32]	Y	NA	NA	Y	NA	NA	NA	N	Y	Y	4
Lee 2018 [40]	Y	Y	Y	Y	NA	NA	NA	N	Y	Y	6
Liu 2014 [46]	Y	N	NA	NA	NA	NA	NA	N	Y	Y	3
Liu 2010 [28]	Y	Y	Y	Y	NA	NA	NA	N	Y	Y	6
Najafi 2017 [47]	Y	Y	Y	Y	NA	NA	NA	N	N	Y	5
Ranjithkumar 2013 [24]	Y	NA	NA	Y	NA	NA	NA	N	Y	Y	4
Rashedinia 2020 [41]	Y	Y	NA	NA	NA	NA	NA	N	Y	Y	4
Sharma 2006 [33]	Y	NA	NA	Y	NA	NA	NA	N	Y	Y	4
Suryavanshi 2020 [34]	Y	Y	Y	Y	NA	NA	NA	N	Y	Y	6
Tian 2016 [35]	Y	Y	Y	NA	NA	NA	NA	N	Y	Y	5
Tiwari 2011 [27]	Y	Y	Y	NA	NA	NA	NA	N	Y	Y	5
Yang 2015 [42]	Y	Y	Y	NA	NA	NA	NA	N	Y	Y	5
Zhou 2012 [22]	Y	Y	Y	Y	NA	NA	NA	N	N	Y	5

## Data Availability

Data for the screening and other datasets used in this study are available on request from corresponding authors.
